# Multifractality through Non-Markovian Stochastic Processes in the Scale Relativity Theory. Acute Arterial Occlusions as Scale Transitions

**DOI:** 10.3390/e23040444

**Published:** 2021-04-09

**Authors:** Nicolae Dan Tesloianu, Lucian Dobreci, Vlad Ghizdovat, Andrei Zala, Adrian Valentin Cotirlet, Alina Gavrilut, Maricel Agop, Decebal Vasincu, Igor Nedelciuc, Cristina Marcela Rusu, Irina Iuliana Costache

**Affiliations:** 1 Cardiology Department, “Sf. Spiridon” University Hospital, 700111 Iasi, Romania; dan_tesloianu@yahoo.com (N.D.T.); ii.costache@yahoo.com (I.I.C.); 2 Department of Physical and Occupational Therapy, “VasileAlecsandri” University of Bacau, 600115 Bacau, Romania; luciandd@yahoo.com (L.D.); spitalmoinesti@bacau.astral.ro (A.V.C.); 3 Biophysics and Medical Physics Department, Faculty of Medicine, “Grigore T. Popa” University of Medicine and Pharmacy, 16 University Str., 700115 Iasi, Romania; vlad.ghizdovat@umfiasi.ro; 4 Municipal Emergency Hospital Moineşti, 1 Zorilor Street, 605400 Moinești, Romania; andrei_zala@yahoo.com; 5 Faculty of Mathematics, “Alexandru Ioan Cuza” University, Carol I Bd., No. 11, 700506 Iasi, Romania; gavrilut@uaic.ro; 6 Physics Department, “Gheorghe Asachi” Technical University, Prof. dr. docent Dimitrie Mangeron Rd., No. 59A, 700050 Iasi, Romania; crina.rusu@yahoo.com; 7 Academy of Romanian Scientists, 54 Splaiul Independentei, Sector 5, 050094 Bucuresti, Romania; 8 Biophysics Department, Faculty of Dental Medicine, “Grigore T. Popa” University of Medicine and Pharmacy, 16 University Str., 700115 Iasi, Romania; decebal.vasincu@umfiasi.ro; 9 Institute of Cardiovascular Disease “G.I.M. Georgescu”, 700503 Iasi, Romania; igor.nedelciuc@yahoo.com

**Keywords:** multifractality, non-Markovian stochastic process, scale relativity theory, Bingham fluid, acute arterial occlusion

## Abstract

By assimilating biological systems, both structural and functional, into multifractal objects, their behavior can be described in the framework of the scale relativity theory, in any of its forms (standard form in Nottale’s sense and/or the form of the multifractal theory of motion). By operating in the context of the multifractal theory of motion, based on multifractalization through non-Markovian stochastic processes, the main results of Nottale’s theory can be generalized (specific momentum conservation laws, both at differentiable and non-differentiable resolution scales, specific momentum conservation law associated with the differentiable–non-differentiable scale transition, etc.). In such a context, all results are explicated through analyzing biological processes, such as acute arterial occlusions as scale transitions. Thus, we show through a biophysical multifractal model that the blocking of the lumen of a healthy artery can happen as a result of the “stopping effect” associated with the differentiable-non-differentiable scale transition. We consider that blood entities move on continuous but non-differentiable (multifractal) curves. We determine the biophysical parameters that characterize the blood flow as a Bingham-type rheological fluid through a normal arterial structure assimilated with a horizontal “pipe” with circular symmetry. Our model has been validated based on experimental clinical data.

## 1. Introduction

Blood, just as the majority of biological fluids, is a “mysterious one.” This is due to the highly complicated structures of the blood, which change depending on health and life conditions [1]. From a physical perspective, blood is a viscoelastic, complex fluid. The notion of a “complex fluid” usually means a non-Newtonian fluid, which means that the shear stress and rate of strain are not directly proportional. The non-Newtonian trait of blood is mainly given by the various cells in it (usually making up 45% of the blood’s total volume), which makes blood a suspension of particles [2,3]. When blood circulates, the particles (cells) interact with plasma and among each other.

Blood rheology and modelling, fundamental issues concerning blood, still need a lot of study. Extracting data from the rheological parameters of blood could help with the diagnosis of medical disorders. In addition, a good understanding of these parameters is necessary for the mathematical modelling of blood flow and devising blood circulation equations.

However, blood modelling and rheological analysis is still incomplete [1]. Blood is a highly concentrated, complex suspension of polydisperse cells. These cells are flexible and have chemical and electrostatic activity. The cells are suspended in an electrolyte fluid (plasma) of critical pH in which numerous proteins and organic substances can be found.

As the motions of a concentrated suspension have a high degree of complexity, no comprehensive theory has been developed to describe the flow of a general multi-component system, blood included. Moreover, the shapes of this type of fluid’s interfaces are convoluted and fragmented, thereby making the application of classical numerical models almost impossible (e.g., finite difference or finite element methods) [1].

The complex behavior of blood and its interactions with the blood vessels’ walls have crucial roles in the physiology of blood flow. Blood interacts mechanically and chemically with the vessel walls, which can get deformed by the action of blood pressure [4].

The usual models used to describe complex fluid dynamics (biological fluids, polymers, foams, etc.) are based on a combination of fundamental theories, which were usually derived from physics and computer models [5,6,7,8]. If the description of the complex fluid dynamics implies computational simulations based on specific algorithms [8,9,10], then their developments related to standard physics theories rely on various classes of models:

(i) Based on the usual conservation laws, developed on spaces with integer dimensions, i.e., the ones from the differentiable class of models (differentiable models) [5,6,7];
(ii) Based on conservation laws, developed in spaces with non-integer dimensions and explicitly written through fractional derivatives, i.e., the ones from the non-differentiable class of models (fractal or multifractal models) [9,10].

Recently, a new class of models is being developed, based on scale relativity theory, either with the monofractal dynamics as in the case of Nottale [11], or with the multifractal dynamics as in the case of the multifractal theory of motion [12].

In the context of scale relativity theory (in Nottale’s sense [11]), as well as in the context of the multifractal theory of motion [12], supposing that any complex fluid dynamics are structurally and functionally assimilated into a multifractal object, these dynamics can be described through motions of the complex fluid’s structural units (dependent on the chosen scale resolution) on continuous and non-differentiable curves (multifractal curves). Since for a large temporal scale resolution with respect to the inverse of the highest Lyapunov exponent [13,14], the deterministic trajectories of any structural units composing a complex system can be replaced by a collection of potential (“virtual”) trajectories, the concept of definite trajectory can be substituted by the one of probability density.

Then, multifractality expressed through stochasticity becomes operational in the description of the dynamics of any complex fluid. This means that, instead of operating with a single variable described by a strictly non-differentiable function, it is possible to operate only with approximations of this mathematical function, obtained by averaging them on different scale resolutions. Therefore, any variable aiming to describe complex fluid dynamics will perform as the limit of a family of mathematical functions, being non-differentiable for null scale resolutions and differentiable otherwise [11].

In the present paper an extension of Nottale’s theory is given, which is based on multifractalization through non-Markovian stochastic processes. Some consequences of this model are explicated in the form of acute arterial occlusions as scale transitions.

## 2. Multifractal Conservation Laws

Let us make the assumption that any complex fluid can be assimilated into a multifractal object. Then, its dynamics (in the multifractal theory of motion) are described through continuous but non-differentiable curves (multifractal curves). This leads us to the following consequences [11,12]:

(i) The lengths of multifractal curves tend to infinity when the scale resolution *δt* tends to zero (according to the Lebesgue theorem [11]). Therefore, the space of such dynamics becomes a multifractal in Mandelbrot’s sense [15].
(ii) During the zoom operation of δt, any dynamics are related to the behaviors of a set of functions through the substitution principle *δt* ≡ *dt*.
(iii) Any dynamics are described by multifractal functions. Then, two derivatives can be defined:
  (1) $$\begin{array}{c} \frac{dQ_{+}}{dt}=\underset{\Delta t\to 0}{\lim}\frac{Q\left(t,t+\Delta t\right)-Q\left(t,\Delta t\right)}{\Delta t}\;\\ \frac{dQ_{-}}{dt}=\underset{\Delta t\to 0}{\lim}\frac{Q\left(t,\Delta t\right)-Q\left(t-\Delta t,\Delta t\right)}{\Delta t} \end{array}$$
  The sign “+” refers to the forward dynamics of the complex fluid. The sign “−” refers to the backward dynamics of the complex fluid.
(iv) The differential of the spatial coordinates has the form:
  (2) $$d_{\pm }X^{i}\left(t,\;dt\right)=d_{\pm }x^{i}\left(t\right)+d_{\pm }\xi \left(t,\;dt\right)$$
  The differentiable part $d_{\pm }x^{i}\left(t\right)$ does not depend on the scale resolution, while the non-differentiable part $d_{\pm }\xi \left(t,\;dt\right)$ is scale resolution dependent.
(v) The quantities $d_{\pm }\xi \left(t,\;dt\right)$ satisfy the relation:
  (3) $$d_{\pm }\xi^{i}\left(t,\;dt\right)=\lambda_{\pm }^{i}{\left(dt\right)}^{\left[\frac{2}{f\left(\alpha \right)}\right]-1},\;f\left(\alpha \right)=f\left[\alpha \left(D_{F}\right)\right]$$
  where $\lambda_{\pm }^{i}$ are constant coefficients associated with the differential-non-differential transition, $f\left[\alpha \left(D_{F}\right)\right]$ is the singularity spectrum of order α, α is the singularity index and $D_{F}$ is the fractal dimension of the “motion curves.”

The fractal dimension can be defined in many ways. Thus, several fractal dimensions may be employed, but the fractal dimension in the sense of Hausdorff–Besikovitch [15] or the fractal dimension in the sense of Kolmogorov [15], are the most commonly used ones. In the case of many models, selecting one of these definitions and using it in the context of any complex fluid dynamics implies that the value of the fractal dimension must be constant and arbitrary for the entirety of the dynamical analysis: for example, it is regularly found that $D_{F}<2$ for correlative processes in the dynamics of any complex fluid; $D_{F}>2$ for non-correlative processes. In the description of complex fluid’s dynamics, we operate with $f\left[\alpha \left(D_{F}\right)\right]$ (i.e., simultaneously operating with several fractal dimensions, on multifractal manifolds, as in the multifractal theory of motion) instead of operating with $D_{F}$ (i.e., with a single fractal dimension, on monofractal manifolds, as in the case of Nottale’s model). This leads to a series of advantages [13], such as the possibility to identify the areas of complex fluid’s dynamics that are characterized by certain individual fractal dimensions (for example, blood components in healthy or pathological cases) or to identify the number of areas in the complex fluid’s dynamics for which the fractal dimensions are situated in an interval of values (for example, cholesterol particles dynamics from patients with cardiac afflictions). Finally, one of the biggest advantages of the method is the ability to identify classes of universality in the complex fluid’s dynamics, even when regular or strange attractors have various aspects (for example, the diagnosis of some cardiac diseases from regular or strange attractor dynamics [16]).

(vi) The differential time reflection invariance is recovered by means of the operator:
  (4) $$\frac{\hat{d}}{dt}=\frac{1}{2}\left(\frac{d_{+}+d_{-}}{dt}\right)-\frac{i}{2}\left(\frac{d_{+}-d_{-}}{dt}\right)$$
  In such context, applying this operator to $X^{i}$ yields the complex velocity:
  (5) $${\hat{V}}^{i}=\frac{\hat{d}X^{i}}{dt}=V_{D}^{i}-V_{F}^{i}$$
  with
  (6) $${\hat{V}}^{i}=\frac{\hat{d}X^{i}}{dt}=V_{D}^{i}-V_{F}^{i}$$
  In this relation the differential velocity $V_{D}^{i}$ is scale resolution independent, while the non-differentiable one $V_{F}^{i}$ is scale resolution dependent.
(vii) Since the multifractalization describing complex fluids dynamics implies stochasticization, the whole statistic “arsenal” (averages, variances, covariances, etc.) is operational. Thus, for example, let us select the subsequent functionality:
  (7) $$\langle d_{\pm }X^{i}\rangle \equiv d_{\pm }x^{i}$$
  with
  (8) $$\langle d_{\pm }\xi^{i}\rangle =0$$
  We will use in the following such a selection.
(viii) Taking the above into account, the complex fluids dynamics can be described through the scale covariant derivative given by the operator
  (9) $$\frac{\hat{d}}{dt}=\partial_{t}+{\hat{V}}^{i}\partial_{i}+D^{lk}\partial_{l}\partial_{k}$$
  where
  (10) $$D^{lp}=\frac{1}{4}{\left(dt\right)}^{\frac{2}{f\left(\alpha \right)}-1}\left(d^{lp}+i{\bar{d}}^{lp}\right),\;\;i=\sqrt{-1}\;$$
  (11) $$d^{lp}=\lambda_{+}^{l}\lambda_{+}^{p}-\lambda_{-}^{l}\lambda_{-}^{p}$$
  (12) $${\bar{d}}^{lp}=\lambda_{+}^{l}\lambda_{+}^{p}-\lambda_{-}^{l}\lambda_{-}^{p}$$
  (13) $$\partial_{t}=\frac{\partial }{\partial_{t}},\;\;\partial_{l}=\frac{\partial }{\partial X^{l}},\;\;\partial_{l}\partial_{p}=\frac{\partial }{\partial X^{l}}\frac{\partial }{\partial X^{p}},\;\;l,\;p=1,\;2,\;3$$

Now, by accepting the scale covariant principle (in the description of any complex fluid dynamics), the conservation law of the specific momentum (geodesic equations on multifractal manifolds) takes the form:

(14) d^V^idt=∂tV^i+V^l∂iV^i+14(dt)[2f(α)]−1Dlp∂l∂pV^i=0

The explicit form of $D^{lp}$ depends on the type of multifractalization used. It can be admitted, according to the previously presented consequences of non-differentiability, that the multifractalization process can take place through various stochastic processes. Usually, stochastic Markovian (thus memoryless) processes are utilized when describing any complex fluid dynamics (see, in particular, the scale relativity theory in Nottale sense [11], i.e., complex fluids’ dynamics on monofractal manifolds described through fractal curves with $D_{F}\to 2$-Peano-type curves [11]). However, since natural processes exhibit memory-like qualities, it is necessary to employ stochastic non-Markovian processes. In this case, wherein it is possible to generalize many of the previous results [11], the following constraints are admitted:

(15) 14(dt)[2f(α)]−1dlp=αδlp, 14(dt)[2f(α)]−1d¯lp=βδlp

where α and β are two constant coefficients associated with the differentiable–non-differentiable scale transitions, and $\delta^{lp}$ is Kronecker’s pseudotensor. Thus, Equation (14) with the restriction Equation (15) yields:

(16) $$\partial_{t}{\hat{V}}^{i}+{\hat{V}}^{l}\partial_{i}{\hat{V}}^{i}+\left(\alpha +i\beta \right)\partial_{l}\partial^{l}{\hat{V}}^{i}=0$$

After Equation (16), the separation of complex fluid dynamics at various scale resolutions implies either a conservation law for the specific momentum at differentiable scale resolutions,

(17) $$\left(\partial_{t}+V_{D}^{l}\partial_{l}+\alpha \partial_{l}\partial^{l}\right)V_{D}^{i}=\left[V_{F}^{l}\partial_{l}-\beta \partial_{l}\partial^{l}\right]V_{F}^{i},$$

or a conservation law for the specific momentum at non-differentiable scale resolutions,

(18) $$\left(\partial_{t}+V_{D}^{l}\partial_{l}+\alpha \partial_{l}\partial^{l}\right)V_{F}^{i}=\left[V_{F}^{l}\partial_{l}-\beta \partial_{l}\partial^{l}\right]V_{D}^{i}$$

Thus, any geodetic motion on multifractal manifolds (i.e., non-constrained free motions on multifractal manifolds—see Equation (16)) is found as correlated with non-geodetic motions on Euclidean manifolds (i.e., constrained motions on Euclidean manifolds—see Equations (17) and (18)), induced either through a specific multifractal force at differentiable scale resolution,

(19) $$f_{D}^{i}=\left(V_{F}^{l}\partial_{l}-\beta \partial_{l}\partial^{l}\right)V_{F}^{i},$$

or through a specific multifractal force at non-differentiable scale resolution:

(20) $$f_{F}^{i}=\left(V_{F}^{l}\partial_{l}-\beta \partial_{l}\partial^{l}\right)V_{D}^{i}$$

In order to correlate the non-geodetic dynamics on Euclidean manifolds, constraints arising from the multifractal-non-multifractal transition must be exploited. In this case, the velocity field associated with the differentiable–non-differentiable scale transitions (multifractal–non-multifractal scale transitions):

(21) $${\bar{V}}^{l}=V_{D}^{l}-V_{F}^{l}$$

satisfies, by substracting Equations (17) and (18), the conservation law of the relative specific momentum:

(22) $$\left[\partial_{t}+{\bar{V}}^{l}\partial_{i}+\left(\alpha +\beta \right)\partial_{l}\partial^{l}\right]{\bar{V}}^{i}=2\left(V_{F}^{l}\partial_{l}-\beta \partial_{l}\partial^{l}\right)V_{F}^{i}$$

In the case

(23) $$f^{i}=2\left(V_{F}^{l}\partial_{l}-\beta \partial_{l}\partial^{l}\right)V_{F}^{i}\equiv 0$$

and for an incompressible complex fluid

(24) $$\partial_{i}{\bar{V}}^{i}=0$$

the differential Equations (22) and (24) constitute non-stationary Navier–Stokes type systems at differentiable–non-differentiable scale transitions. In the particular case of a stationary Navier–Stokes type system, these differential equations, in dimensionless plane coordinates, with adequate initial and boundary conditions, admit the following solutions [17]:

(25) $$U=\frac{1.5}{{\left(\nu \xi \right)}^{\frac{1}{3}}}{\sec h}^{2}\left[\frac{0.5\eta }{{\left(\nu \xi \right)}^{\frac{2}{3}}}\right]$$

(26) $$V=\frac{1.9}{{\left(\nu \xi \right)}^{\frac{1}{3}}}\left\{\frac{\eta }{{\left(\nu \xi \right)}^{\frac{2}{3}}}{\sec h}^{2}\left[\frac{0.5\eta }{{\left(\nu \xi \right)}^{\frac{2}{3}}}\right]-\tan h\left[\frac{0.5\eta }{{\left(\nu \xi \right)}^{\frac{2}{3}}}\right]\right\}$$

where ξ and η are nondimensional spatial coordinates; U and V are the nondimensional components of the velocity field along the Oξ and Oη axes; and ν is the multifractality degree.

Therefore, the velocity field along the Oξ axis is described by the multifractal soliton Equation (25), while the velocity field along the Oη axis is described by the multifractal soliton—kink Equation (26). For details on the nonlinear classical solution of soliton and kink types, see [13,14].

In such a context, when investigating the dynamics of a complex fluid’s expansion in a multifractal medium, there are two types of scales that need to be considered. Firstly, there are the internal interaction scales, which are amalgams of dynamics induced by the properties of the complex fluid and by its nature. Secondly, the external interaction scales contain the dynamics between the complex fluid and the multifractal medium in which the fluid is embedded.

**Figure 1:**
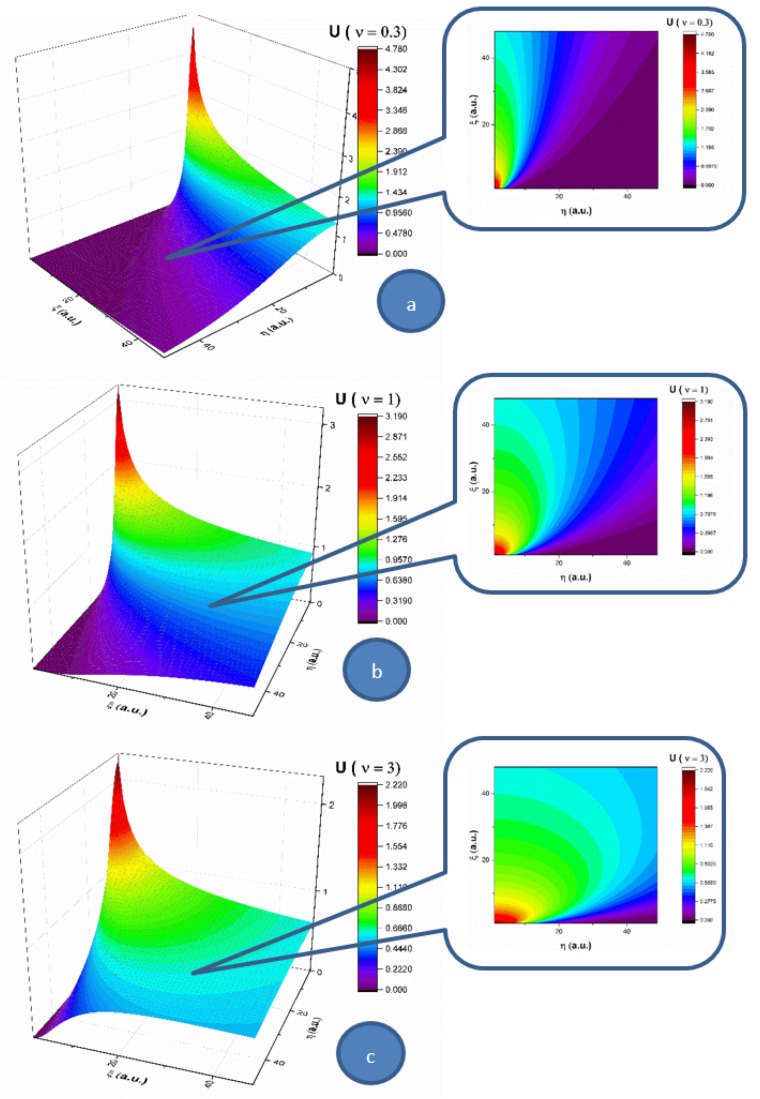
(**a**–**c**). 3D and contour plot representations of the velocity component on the *Oξ* for three multifractality degrees: (**a**) 0.3; (**b**) 1; and (**c**) 3. The velocity increases from purple to red.

**Figure 2:**
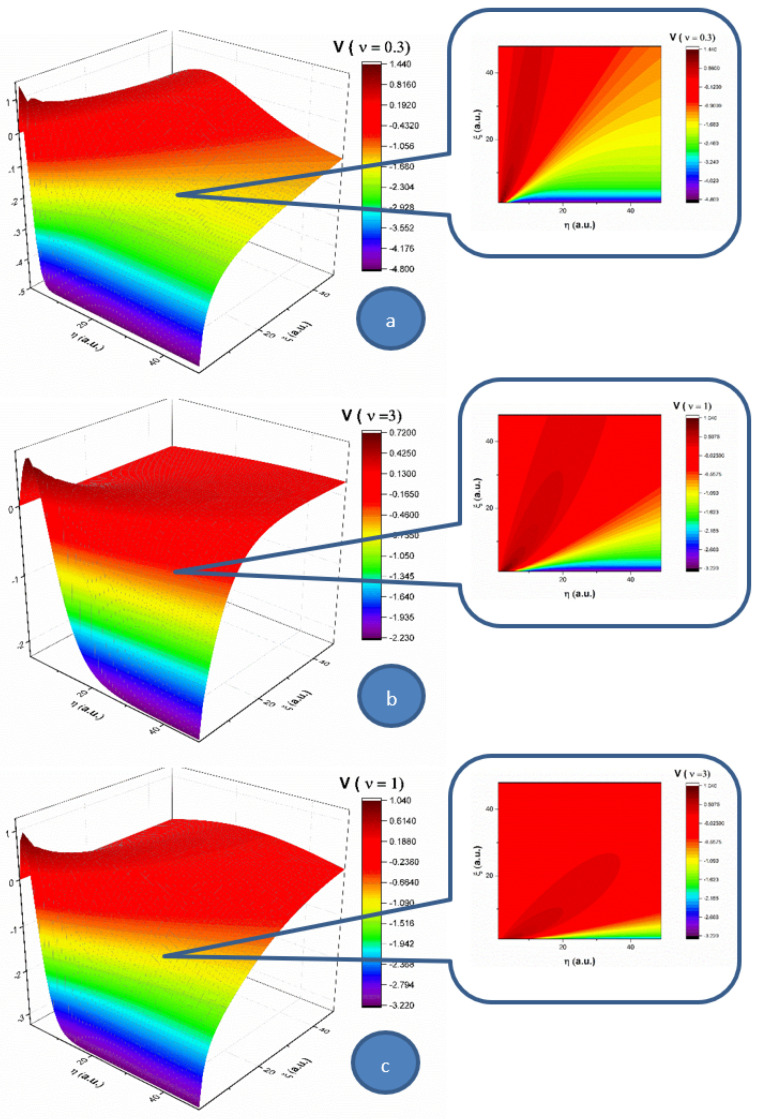
3D and contour plot representations of the velocity component on the *Oη* for three multifractality degrees: (**a**) 0.3, (**b**) 1 and (**c**) 3. The velocity increases from purple to red.

In the following, let us analyze the influence of the multifractality degree on each of the two components (*U* and *V*) of the complex fluid, for a 2D flow. In Figure 1, the velocity component (*U*) of *Oξ*, for three multifractality degrees (0.3, 1 and 3), is presented in 3D and contour plots. For a low multifractality degree we can notice a very directional flow mainly across the *Oξ*, with little spatial expansion. An increase in multifractality in the system leads to a decrease of the velocity and a strong lateral expansion. It is important to note that the main expansion direction does not change; only the contributions in *Oη* direction do. The multifractality degree of the system on this velocity component acts as a multifractal-like dispersion phenomenon. In Figure 2, the velocity component (*V*) of *Oη* for three multifractality degrees (0.3, 1 and 3) is presented in 3D and contour plots. When investigating the absolute value of the velocity, this component of the velocity is not influenced by the multifractality degree, thereby remaining quasi constant. There is however a strong influence on the direction of the component. For a low multifractality degree, there is a small angle with respect to the *Oξ* axis. Higher values of multifractality induce a change in the expansion angle, transitioning towards higher angles. The multifractality degree of the complex fluid on this velocity component acts towards the uniformization of the *V* component, as the distribution tends to reach the maximum expansion velocity available for the complex fluid.

By extrapolating these results in the case of blood flow in the circulatory system, an explanation for low density lipoprotein (LDL) cholesterol deposition on blood vessels’ walls can be given (due to the fact that, having a lower velocity at the wall, LDL can penetrate the intima). Moreover, in pathological cases where LDL values are high, corresponding thus to a high fractality degree, according to the same dynamics (an increase in multifractality induces an expansion towards the wall), the probability of LDL deposition on blood vessels’ walls increases [2,3].

Let us remember the fact that, in the case of blood’s laminar flow (with constant velocities of blood’s components), taking into account the average dimensions of LDL and HDL cholesterol (18–25 nm for LDL and 5–12 nm for HDL), LDL particles move slower with respect to HDL particles. Consequently, LDL can “accumulate” in the intima, while HDL has a different behavior [2,3].

Let us note that a multifractal minimal vortex can be associated with the velocity field given through Equations (25) and (26):

(27) $$\begin{array}{l} \Omega =\left(\partial_{\eta }U-\partial_{\xi }V\right)\\ \;=\frac{0.57\eta }{{\left(\nu \xi \right)}^{2}}+\frac{0.63\xi }{{\left(\nu \xi \right)}^{\frac{4}{3}}}\tan h\left[\frac{0.5\eta }{{\left(\nu \xi \right)}^{\frac{2}{3}}}\right]+\frac{1.9\eta }{{\left(\nu \xi \right)}^{2}}{\sec h}^{2}\left[\frac{0.5\eta }{{\left(\nu \xi \right)}^{\frac{2}{3}}}\right]--\frac{0.57\eta }{{\left(\nu \xi \right)}^{2}}{\tan h}^{2}\left[\frac{0.5\eta }{{\left(\nu \xi \right)}^{\frac{2}{3}}}\right]\\ \;-\left[\frac{1.5}{\nu \xi }+\frac{1.4\eta }{\xi {\left(\nu \xi \right)}^{\frac{5}{3}}}\right]{\sec h}^{2}\left[\frac{0.5\eta }{{\left(\nu \xi \right)}^{\frac{2}{3}}}\right]\tan h\left[\frac{0.5\eta }{{\left(\nu \xi \right)}^{\frac{2}{3}}}\right] \end{array}$$

**Figure 3:**
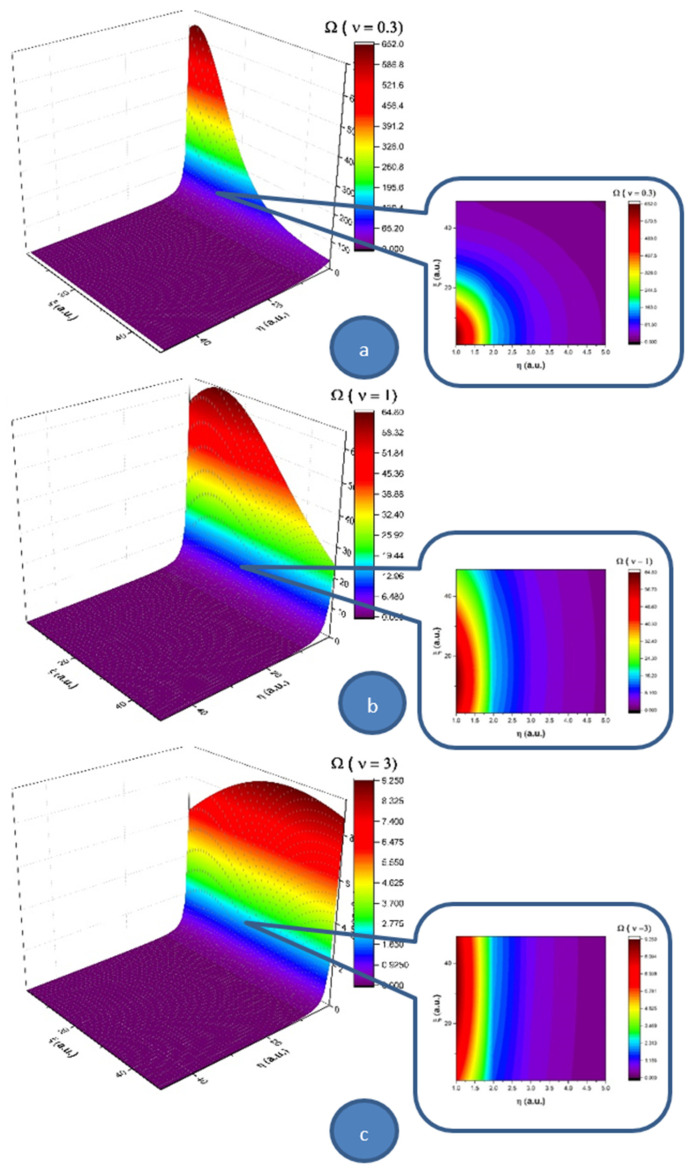
3D and contour plot representations of the multifractal minimal vortex for three multifractality degrees: (**a**) 0.3, (**b**) 1 and (**c**) 3. The vortex field increases from purple to red.

In Figure 3, we present 3D and contour plots of the multifractal minimal vortex.

Thus, the multifractal soliton Equation (24) and the soliton-kink multifractal mixture Equation (25) are responsible, through the multifractal minimal vortex Equation (27), for turbulences management at non-differentiable scale resolutions. Although they are non-manifested at differentiable scale resolutions, these turbulences can become manifest at the same scale through the “synchronization” (self-structuring) of multifractal minimal vortices in the form of vortices streets. As we will show in the following, such a self-structuring can be responsible, for example, in the case of blood as a complex fluid, for thrombus generation with serious implications for arterial occlusions. This self-structuring can be assimilated into the differentiable–non-differentiable scale transition, a situation in which the specific multifractal force Equation (23) must be identified with a pressure gradient in the form

(28) $$f_{D}^{i}=\left(V_{F}^{l}\partial_{l}-\beta \partial_{l}\partial^{l}\right)V_{F}^{i}=-\frac{1}{\rho }\partial^{i}p$$

## 3. Model Application for Blood as a Complex Fluid. Arterial Occlusions as a Result of the Differentiable-Non-Differentiable Scale Transition

An acute arterial occlusion (peripheral vessels or coronary artery) of an artery that has no significant pre-existent lesions leads to dramatic consequences due to the lack of collateral substitutive circulation, as this kind of circulation usually develops within years, in the presence of hemodynamic significant stenosis [18,19].

Classical biophysical models which explain this phenomenon take into account the cracking of an intimal atheroma plaque, which is hemodynamic insignificant, the activation of the pro-thrombogenic cascade through the denudation of the endothelium and the formation in certain circumstances (the nature of this circumstances is, however, not clearly stated, making us discuss the not very scientific term “bad luck”) of a completely occlusive thrombus [20,21,22]. At least one counterargument should be taken into consideration due to its simplicity: why does an occlusive thrombus form so quickly in the absence of a stenosis, when the sanguine flux is unaltered? Why does the “wash-out’’ phenomenon not appear?

### 3.1. Premises and Purposes

Without contradicting the above stated theory, which is sustained by some morph-pathological evidence, we will prove through a biophysical fractal model that the blocking of the lumen of an absolutely healthy artery can happen as a result of the “stopping effect” (even in the absence of—at least disputably—the cracked and non-protrusive atheroma plaque), in the conditions of a normal sanguine circulation.

This happens due to the fact that blood is a complex non-Newtonian fluid made of plasma and formed cells, cholesterol vesicles and other suspended elements [23]; thus, the laws of fractal physics are completely applicable to sanguine circulation. For conformity, the perfect Newtonian fluid is a fluid in which viscosity is independent of the shear stress, thus having no relation to the sanguine fluid. However, not only does the complex structure of blood justify the use of fractality, but so does the complex structure of the arterial system, with its multiple ramifications, which generate turbulence areas and interruptions of the linear flowing that make classical physics not applicable in this context. We actually discuss multifractality: a morphological one due to complex structure of the arterial tree as well as a functional one due to blood flow “regimes.”

Fractal physics and mathematics (“of non-integral objects”) conceptually defined by Benoît Mandelbrot [15] manages to explain the complex biophysical phenomena by accepting and defining the permanent interrelations between the components of a structural unit: organ/organism.

Hence, fluids with nonlinear viscous behaviors and viscoelastic materials are complex fluids [1]. A great variety of materials can be assimilated into complex fluids: polymers (elastomers, thermoplastics and composites), biological fluids (DNA, which creates cells by means of simple but very elegant language and it is responsible for the remarkable way in which individual cells organize into complex systems, such as organs; and these organs form even more complex systems, such as organisms, proteins, cells, dispersions of biopolymers and cells and human blood), colloidal fluids, foams, suspensions, gels, emulsions, micellar and liquid-crystal phases and molten materials.

Standard theoretical biophysical models normally used in complex fluid dynamics, and particularly those of blood flow through blood vessels, are ambiguous. The assessment that the entities contained by blood move along continuous and differentiable curves proves to be false, as it cannot comprise the entire variety of dynamics that are induced by the flowing of blood through the circulatory system (from the separation of blood components through turbulence regimes to blood–blood vessel interactions).

In this context, the hypothesis according to which blood entities move on continuous but non-differentiable curves (and particularly on fractal/multifractal curves) becomes natural, and moreover, lacks any contradictions. We cannot predict the entirety of the blood vessel system interactions, blood–organic tissue interactions, etc., or at an elementary level, blood entity-blood entity (lymphocyte-granulocyte, etc.) interactions. That is why accepting the above-stated hypothesis is a simple, elegant and efficient solution, with the impossibility of predicting all these interactions that take place being substituted by the use of fractality/multifractality [6].

We are thus concentrated to the dynamics of a special type of fluid, free of interactions, in which the streamlines are continuous and non-differentiable curves (denoted from this point on as a fractal/multifractal fluid). When analyzing these types of dynamics, the scale relativity theory (SRT), in any of its forms, becomes operational.

In the present section, we propose a biophysical fractal/multifractal model in the form of a SRT approach to analyze the blood flow dynamics. Particularly, we determine the biophysical parameters that characterize the blood flow as a Bingham-type rheological fluid through an abnormal arterial structure assimilated with a horizontal “pipe” with circular symmetry, under the action of a pressure gradient induced by ventricular inotropic force as well as by the arterial wall elasticity for a given site. The study contains an analytical solution using fractal/multifractal Navier-Stokes-type equations with cylindrical symmetry in the SRT approach. Our model differs than other models used to describe Bingham-type rheological fluids.

### 3.2. Blood Behaviors as a Bingham-Type Rheological Fluid

Biological fluids that have a fluid continuous phase can also have a discontinuous phase given by solid or fluid particles with different properties, such as density, granulometry and shape. The discontinuous phase of these biological fluids, in different working conditions (depositing, transport or phase separation), do not have unitary behavior that could exactly be characterized [5,6]. In the majority of cases, the discontinuous phases in a biological fluid with laminar flow through a circular pipe or linear flow with flow direction change (bends, velocity limiters and reductions) concentrate towards the axis of the pipe with a distribution that is proportional to the size particle. Therefore, these fluids are complex molecular structures that do not obey Newton’s law, which are called rheological fluids (complex fluids) [5,6]. Bingham fluids are included in this category.

**Figure 4:**
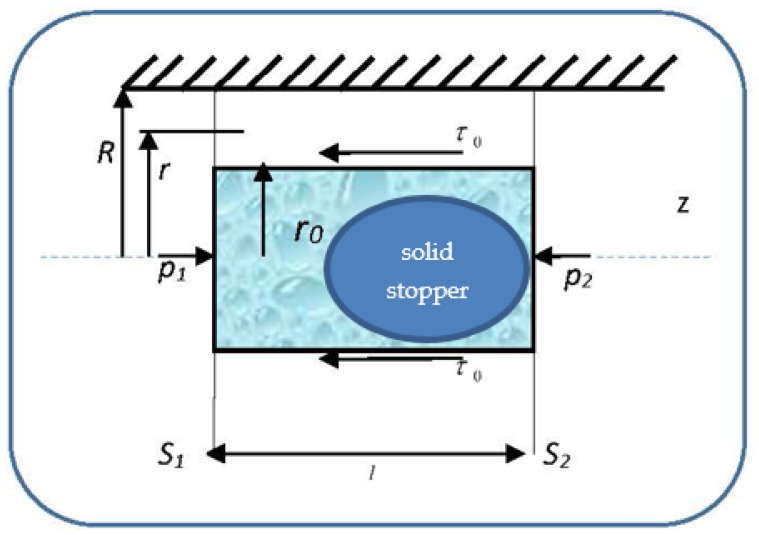
Pressure gradient flow induced by ventricular inotropic force as well as by the arterial wall (hatched area) elasticity for a given zone of the blood as a Bingham-type rheological fluid through a normal arterial structure assimilated with a circular pipe. *l*—the length of the stopper; *S*_1_ and *S*_2_—the lateral surfaces of the solid stopper; *p*_1_ and *p*_2_—the pressures along the solid stopper; *R*—the radius of the artery; *r*_0_—the radius of the solid stopper; *r*—a specific distance along which the velocity gradient field is manifested; $\tau_{0}$—deformation tangential unitary effort; *z*—the flow direction.

**Figure 5:**
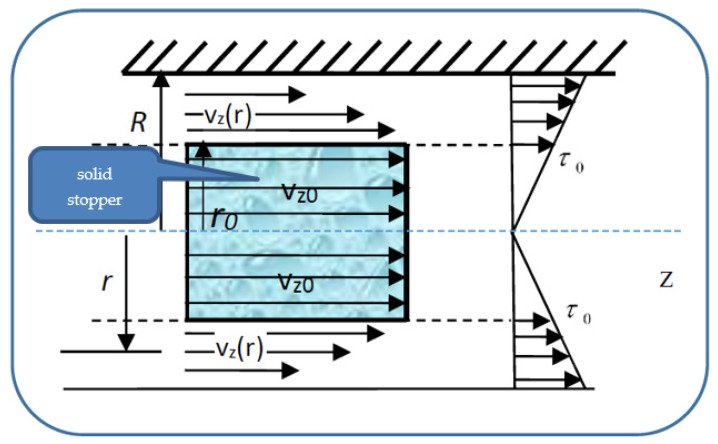
Velocity and viscosity tangential unitary effort diagrams of the blood that flows in an elastic arterial wall. *R*—the radius of the artery; *r*_0_—the radius of the solid stopper; *r*—a specific distance along which the velocity gradient field is manifested; $\tau_{0}$—deformation tangential unitary effort; *z*—the flow direction; v_z0_—the velocity of the solid stopper (blood moves as an apparently undistorted rigid system); v_z_(r)—the velocity of the blood (normal flow).

In such context, the flow properties of blood as a Bingham-type rheological fluid differ from those of blood as a Newtonian fluid. Indeed, the viscosity tangential unitary effort variation law in the blood has the expression

(29) $$\tau =\tau_{0}+\eta \frac{dv}{dr},$$

so that the velocities’ distribution field in a normal arterial structure assimilated with a horizontal pipe with circular symmetry covers two sub-domains. We present in Figure 4 the pressure gradient flow induced by the ventricular inotropic force as well as by the arterial wall elasticity for a given zone of the blood as a Bingham-type rheological fluid through a normal arterial structure assimilated with a circular pipe; in Figure 5, the velocity and viscosity tangential unitary effort diagrams of the blood that flows in an elastic arterial wall are shown.

In relation (29), $\tau_{0}$ is the deformation tangential unitary effort, dv/dr is the blood velocity gradient with respect to the normal on the transversal section and η is the blood viscosity coefficient assimilated into the multifractal degree.

The restriction $\tau_{0}=0$ in Equation (29) describes the blood behaviors as a Newtonian fluid:

(30) $$\tau_{0}=\eta \frac{dv}{dr}$$

In subdomain $0\leq r\leq r_{0}$, the viscosity tangential unitary effort, τ, is lower in value than the deformation tangential unitary effort $\tau_{0}$. As a consequence, blood as a Bingham-type rheological fluid moves as an apparently undistorted rigid system. The solid stopper flows with constant velocity in the central area of the artery, without changing its structure.

In subdomain $r_{0}\leq r\leq R$, the viscosity tangential unitary effort,τ, exceeds the deformation tangential unitary effort $\tau_{0}$. As a consequence, blood as a Bingham-type rheological fluid flows so that layers with finer “particles” and various concentrations appear. We note that the radius $r_{0}$ and the two sub-domains border depending on the rheological characteristic of the blood as a Bingham-type rheological fluid. The mathematical model used in the blood flow dynamics is presented in the following.

### 3.3. Mathematical Procedure

The momentum Equation (29) with constriction Equation (39), together with the continuity type Equation (24) allow the solving of movement problems of biological fluids if the limit conditions are known.

Let us consider the unidirectional flow of blood through a cylindrical blood vessel, with radius *R*, under the action of a pressure gradient.

Under these circumstances, the velocity field Equation (10) is ${\bar{V}}^{l}\equiv \left(v_{r}=0,\;v_{\varphi }=0,\;v_{z}\neq 0\right)$. Moreover, if we use the continuity equation in the imposed conditions, the dynamic equilibrium equation is:

(31) $$\eta \frac{d^{2}v_{z}}{dr^{2}}+\frac{1}{r}\frac{dv_{z}}{dr}=\frac{dp}{dz}$$

Considering the expression of the friction effort for blood, we get:

(32) $$\frac{\partial \tau }{\partial r}+\frac{\tau }{r}=\frac{\partial p}{\partial z}$$

Let us calculate the relations of flowing velocity in the two areas of the blood’s flow regime, under the action of the pressure gradient, through the boundary conditions, both for the flowing velocity $v_{z}$ and the shear velocity *d*v*_z_/dr*.

The solution of Equation (31) is:

(33) $$v_{z}\left(r\right)=\frac{\Delta p}{4\cdot \eta \cdot l}r^{2}+\frac{\tau_{0}}{\eta }r+a\ln r+\;b$$

where a and b are integration constants. The values of these constants are established by the following boundary conditions:

(i) For $r=r_{0}$, i.e., on the stopper borderline, *d*v*_z_/dr* = 0, so we will have:
  (34) $$\frac{dv_{z}}{dr}=\frac{2\cdot r\cdot \Delta p}{4\cdot \eta \cdot l}+\frac{\tau_{0}}{\eta }+\frac{a}{r}$$
  (35) $$\frac{r_{0}^{2}\cdot \Delta p}{2\cdot \eta \cdot l}+\frac{\tau_{0}\cdot r_{0}}{\eta }+a=0$$
  (36) $$a=-\frac{\Delta p}{2\cdot \eta \cdot l}r_{0}^{2}-\frac{\tau_{0}}{\eta }r_{0}$$
  (37) $$v_{z}\left(r\right)=\frac{\Delta p}{4\cdot \eta \cdot l}r^{2}+\frac{\tau_{0}}{\eta }r-\frac{\Delta p}{2\cdot \eta \cdot l}r_{0}^{2}\ln r+\frac{\tau_{0}}{\eta }r_{0}\ln r+b$$
(ii) For r=R,  i.e., at the vessel wall, $v_{z}\left(R\right)=0$.
  (38) $$\frac{\Delta p}{2\cdot \eta \cdot l}\left[\frac{R^{2}}{2}-r_{0}^{2}\ln R\right]+\frac{\tau_{0}}{\eta }\left[R-r_{0}\ln R\right]+b=0$$
  (39) $$b=-\frac{\Delta p}{2\cdot \eta \cdot l}\left[\frac{R^{2}}{2}-r_{0}^{2}\ln R\right]-\frac{\tau_{0}}{\eta }\left[R-r_{0}\ln R\right]$$

The velocity $v_{z}\left(r\right)$ for $r\in \left[r_{0};R\right]$ has the following expression:

(40) $$v_{z}\left(r\right)=\frac{\Delta p}{2\cdot \eta \cdot l}\left[\frac{R^{2}}{2}-\frac{r^{2}}{2}+r_{0}^{2}\ln\frac{R}{r}\right]+\frac{\tau_{0}}{\eta }\left[R-r+r_{0}\ln\frac{R}{r}\right]$$

In order to determine the radius $r_{0}$ of the stopper, we take into account a cylinder of radius $r_{0}$ placed inside the vessel, which is at equilibrium under pressure and shear forces’ actions.

From the dynamic equilibrium equation of pressure and friction forces on the stopper (of radius *r*_0_ and length *l*), i.e.,

(41) $$\left(p_{1}-p_{2}\right)\pi r_{0}^{2}=2\tau_{0}r_{0}\pi l$$

for the radius $r_{0\;}$ of the fluid stopper, the following expression results:

(42) $$r_{0}=\frac{2\tau_{0}l}{\Delta p},\Delta p=p_{1}-p_{2}$$

The movement velocity of the fluid stopper is obtained by imposing in relation Equation (33) the condition $r=r_{0}$. We find the relation:

(43) $$v_{0z}\left(r\right)=\frac{\Delta p}{2\cdot \eta \cdot l}\left[\frac{R^{2}}{2}-\frac{r_{0}{}^{2}}{2}+r_{0}^{2}\ln\frac{R}{r_{0}}\right]+\frac{\tau_{0}}{\eta }\left[R-r_{0}+r_{0}\ln\frac{R}{r_{0}}\right]$$

Let us note that the previous results are extensions of the ones from [24,25], in the sense that blood has been assimilated into a multifractal object, both structurally and functionally.

## 4. Results

Our biophysical fractal model was used for in vivo analyses of 15 clinical cases of patients with an acute occlusive thrombus on an absolutely healthy artery. These cases were selected during a 2-year period (2018–2020). Patients with atrial fibrillation were excluded for preventing mismatch with thromboembolic acute coronary occlusion. Patients with patent foramen ovale (transoesophageal echocardiography study performed) were excluded in order to avoid a paradoxical coronary embolism. IVUS (intravascular ultrasound) or coronary angio-CT were not performed for these patients; even if some irregularities could be seen via angiography, it was clear that there were no significant ulcerated atheroma plaques or major signs of parietal atherosclerosis. Additionally, in patients older than fifty, an absolutely normal coronary wall is probably a fantasy. We had EKG holter monitoring in all patients for exclusion of paroxysmal atrial fibrillation.

We present here the three most relevant cases, with thrombus dimensions of fourty or more millimeters cubed (for the other 12 cases, the thrombus dimensions were between 20 and 60 mm^3^). For all the cases, our theoretical results were verified by coronarography images. Images were obtained by courtesy of the Interventional Catheterization Laboratory, Institute of Cardiovascular Disease “G.I.M. Georgescu” Iasi).

**Figure 6:**
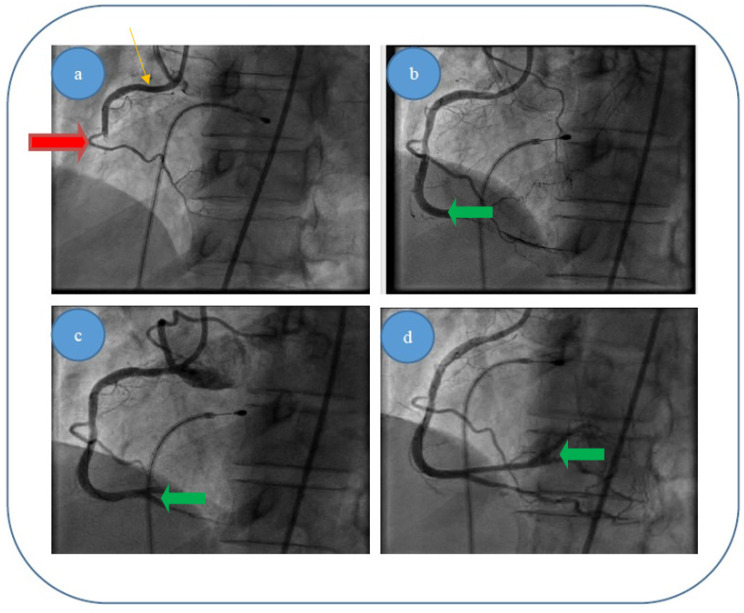
(**a**–**d**). Acute thrombus formation (red arrow) in an apparently healthy artery (orange arrow) with no evidence of plaque dissection—different interventional approach stages for patient 1: (**a**) blood flow before thromboaspiration; (**c**,**d**) blood flow returning to normal after thromboaspiration/removal of thrombus (green arrow). (**b**) Explanation.

**Figure 7:**
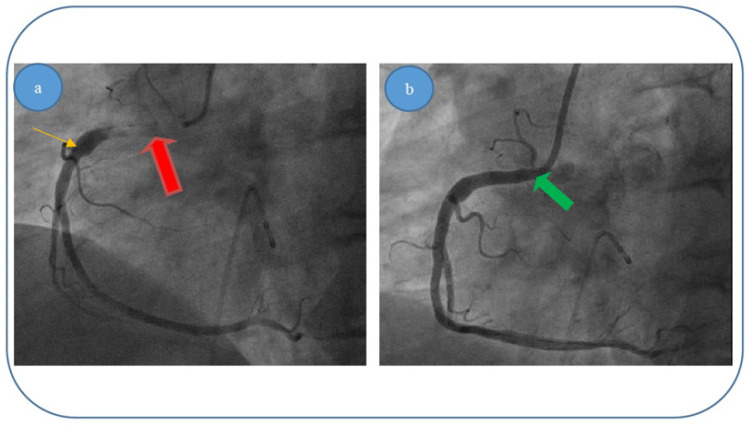
(**a**,**b**). Acute thrombus formation (red arrow) in an apparently healthy artery (orange arrow) with no evidence of plaque dissection—different interventional approach stages for patient 2: (**a**) blood flow before thromboaspiration; (**b**) blood flow returning to normal after thromboaspiration/removal of thrombus (green arrow).

**Figure 8:**
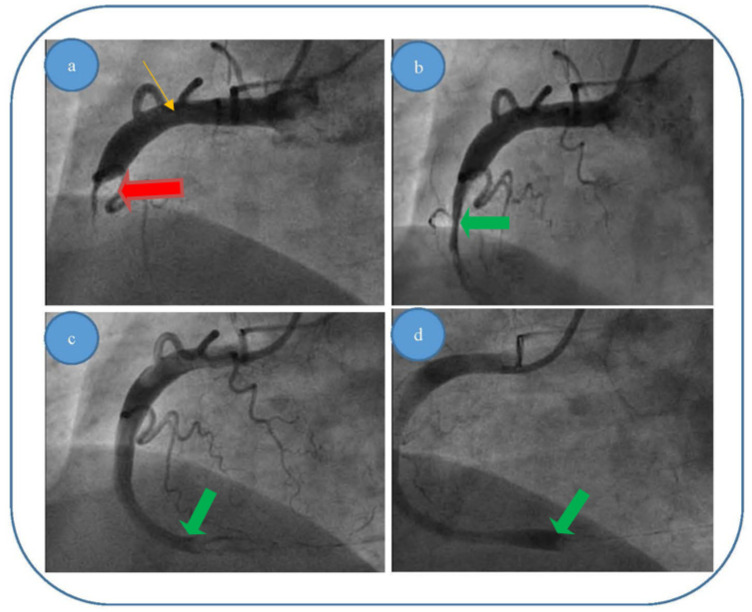
(**a**–**d**). Acute thrombus formation (red arrow) in an apparently healthy artery (orange arrow) with no evidence of plaque dissection—different interventional approach stages for patient 3: (**a**) blood flow before thromboaspiration; (**c**,**d**) blood flow returning to normal after thromboaspiration/removal of thrombus (green arrow). (**b**) Explanation.

(i) Patient 1, a 49 year-old male patient, who was diagnosed with acute infer lateral ischemia; the coronary angiography revealed an acute occlusive thrombus (4–4.5 mm diameter and 60–80 mm length) at the junction between segments I and II of his right coronary artery (belatedly we can observe retrograde loading of the left anterior descending artery) (Figure 6a); after thrombus aspiration, a distal thrombotic embolism appeared with an apparently healthy artery (or possible minimal lesion—no sign of plaque dissection) at the initial thrombus level (Figure 6b); repeated thrombus aspiration at the level of secondary occlusion revealed the posterior descending branch (Figure 6c) and subsequently the posterolateral branch (Figure 6d); finally there is thrombolysis in myocardial infarction (TIMI) 3 flow; also, there was no evident coronary lesion responsible for the above-mentioned pathological phenomena.
(ii) Patient 2, a 67 year-old male patient who was diagnosed with acute inferior, poster lateral and right ventricle ischemia; coronary angiography revealed an acute occlusive thrombus just at the origin of the right coronary artery (5.5–6 mm diameter and approximately 40 mm length); after thrombus aspiration, satisfactory results were obtained with TIMI 3 flow and no evidence of significant atherosclerotic disease at the level of culprit zone was present—see Figure 7a,b.
(iii) Patient 3, a 61 year-old female patient who was diagnosed with acute inferior and poster lateral ischemia; coronary angiography revealed an acute occlusive thrombus extending from the beginning of right coronary artery segment II to crux (4.5–5 mm diameter and approximately 80–100 mm length), possibly with extensions to right posterior descending artery and poster lateral branches; repeated thrombus aspiration with unsatisfying results in terms of distal TIMI flow (0–1), but with no evidence of significant atherosclerotic disease at the level of culprit zone—see Figure 8a–d.

We present in Table 1 the average experimental parameters of blood flow through the right coronary artery, used in our study. We must mention that the values for the average experimental stress as a function of diastolic pressure ($\tau_{0}$), the average experimental diastolic velocity (*v_d_*), the average experimental systolic velocity (*v_S_*), the average experimental blood density (ρ) and the average experimental kinetic viscosity coefficient (η) were not directly measured in vivo, but they were estimated through methods found in specialized literature [26,27].

Taking these into account, the mathematical procedure we developed using our theoretical model had the following steps:

(i) Determining the values of the Reynolds’ number for blood flow through the right coronary artery, using the following relation:
  $$R_{e}=\frac{v_{S}D}{\nu }$$
  where $v_{S}$ is the minimum value of the average experimental systolic velocity of blood, D is the average experimental diameter of the right coronary artery and ν is the average kinetic viscosity coefficient of blood. For
  $$R_{e}<2400$$
  blood flow through the right coronary artery is laminar, while for
  $$R_{e}>2400$$
  blood flow becomes turbulent;
(ii) Determining the values of the loss coefficient of blood flow through the right coronary artery, using Darcy’s coefficient [4]:
  $$\lambda =\frac{64}{R_{e}}=\frac{64\nu }{v_{S}D}$$
(iii) Determining the values of the pressure loss for blood flow through the right coronary artery, using the following relation:
  $$\Delta p=\lambda \frac{L}{D}\rho \frac{v_{d}^{2}}{2}=32\nu \rho \frac{L}{D^{2}}\frac{v_{d}^{2}}{v_{S}}$$
  where L is the average length of the experimental thrombus, ρ is the average experimental blood density and $v_{d}$ is the maximum value of blood’s average experimental systolic velocity;
(iv) Determining the theoretical value of a right coronary artery thrombus, using the relation:
  $$D_{t}=\frac{4\tau_{0}L}{\Delta p}=\frac{1}{8}\frac{v_{S}\tau_{0}D^{2}}{\upsilon \rho v_{d}^{2}}$$
  where $\tau_{0}$ is the average experimental deformation effort of blood. We can see that the thrombus’s theoretical diameter is not dependent on the thrombus’s length, but it is directly proportional to the product of the minimum average experimental systolic blood velocity, the average experimental stress of blood and the square of the average experimental diameter of the right coronary artery; also, it is inversely proportional to the product of the average experimental viscosity coefficient of blood, the average experimental density of blood and the square of the maximum value of the average experimental diastolic velocity of blood.

We present in Table 2 the average theoretical parameters of blood flow through the right coronary artery, obtained using our theoretical model.

We can thus see a good conformity between the values from the theoretical model with the experimental values we found in the three cases presented above. Therefore: (i) for patient 1 we calculated a thrombus diameter of 4.54 mm, while the experimentally measured thrombus diameter was 4 mm; (ii) for patient 2 we calculated a thrombus diameter of 6.82 mm, while the experimentally measured thrombus diameter was 6 mm; (iii) for patient 3 we calculated a thrombus diameter of 5.52 mm, while the experimentally measured thrombus diameter was 5 mm.

## 5. Conclusions

The main conclusions of our paper are the following:

(i) The main results of Nottale’s theory were extended based on multifractalization through non-Markovian stochastic processes. In this context, some specific conservation laws were obtained (specific momentum conservation laws, both at differentiable and non-differentiable resolution scales, a specific momentum conservation law associated with the differentiable–non-differentiable scale transition, etc.).
(ii) From the analysis of the conservation laws, both at differentiable and at non-differentiable resolution scales, we found that the complex fluid’s dynamics are constrained. Eliminating these constraints implies, in the stationary case, both for differentiable and non-differentiable scale resolutions, Navier–Stokes type systems, for which dynamics with plane symmetry can be explicated in the form of multifractal soliton-kink solutions for the velocity field. The presence of such fields implies the “synchronization” (self-structuring) of multifractal minimal vortices in the form of vortices streets. Such self-structuring can be responsible, for example, in the case of blood as a complex fluid, for thrombus generation with serious implications for arterial occlusions.
(iii) In this framework, we prove the existence of the “stopping effect” in a normal arterial portion, an effect which appears through the self-structuring of the normal sanguine flux. The cracking of the atheroma plaque can be integrated in our demonstration, like a trigger of the solid self-structuring on the flowing axis of the complex fluid, even if, as we have proved, this “stopping effect” can appear without any predisposing pathological factor.
(iv) Those points presented above prove the existence of the stopping effect in a cylinder (comparable to a non-ramified arterial portion), an effect which appears through the auto structuring of the normal sanguine flux complex through the normal composition of human blood itself. This happens in the absence of any lesion of the cylinder’s wall. Taking into consideration the temperature and viscosity variations that define the normal functioning of the human body, we can easily understand the phenomenon described. Therefore, this stopping effect which manifests in the artery could be a possible explanation for the premises mentioned at the beginning of our biological: why does an occlusive thrombus form so quickly in the absence of a stenosis, when the sanguine flux is unaltered? Why does the “wash-out’’ phenomenon not appear?
(v) Despite the fact that this theory does not want to annul the classic modelling of the cracking of an atheroma plaque with major thrombosis added in explaining the acute arterial occlusion, we consider that the mathematical modelling offers at least a thoroughly explained and hard to contradict alternative.
(vi) Moreover, our model could offer a plausible explanation for the much discussed but surely proven phenomenon called MINOCA (myocardial infarction with non-obstructive coronary arteries)—an acute occlusion in normally arteries with spontaneous but late thrombus dissolution, with the damage developing despite the normal appearance of the vessels via coronary angiography.

## Figures and Tables

**Table 1:** Average experimental parameters of blood flow through the right coronary artery for the three clinical cases.

Patient	*D_e_*^1^ (mm)	*L*^2^ (mm)	$\tau_{0}{\;}^{3}\;(N/m^{2})$	*v_d_*^4^ (cm/s)	*v_S_*^5^ (cm/s)	ρ^6^ (kg/m^3^)	η^7^ (m^2^/s)
Patient 1	4	70	9/75 mm Hg	35 ± 11	24 ± 7	1060	3.04 × 10^−6^ at 36.5 °C
Patient 2	6	40	6/90 mm Hg	35 ± 11	24 ± 7	1060	3.04 ×10^−6^ at 36.5 °C
Patient 3	5	90	7/90 mm Hg	35 ± 11	24 ± 7	1060	3.04 × 10^−6^ at 36.5 °C
Observations			The method from [26] was used	The method from [26] was used	The method from [27] was used	The method from [27] was used	The method from [26] was used

^1^ Average experimental thrombus diameter. ^2^ Average experimental thrombus length. ^3^ Average experimental stress as a function of diastolic pressure. ^4^ Average experimental diastolic velocity. ^5^ Average experimental systolic velocity. ^6^ Average experimental blood density. ^7^ Average experimental kinetic viscosity coefficient.

**Table 2:** Average theoretical parameters of blood flow through the right coronary artery for the three clinical cases, determined using our theoretical model.

Patient	*R_e_* ^1^	*Λ* ^2^	Δ*p* ^3^ (N/m)	*D_t_*^4^ (mm)
Patient 1	226	0.283	634	4.54
Patient 2	140	0.457	341	6.82
Patient 3	283	0.226	457	5.52

^1^ Reynolds number. ^2^ Darcy’s loss coefficient. ^3^ Pressure loss. ^4^ Thrombus diameter determined using our model.
